# China's space robotics for on-orbit servicing: the state of the art

**DOI:** 10.1093/nsr/nwac129

**Published:** 2022-07-01

**Authors:** XueAi Li, Dapeng Yang, Hong Liu

**Affiliations:** State Key Laboratory of Robotics and Systems, Harbin Institute of Technology, China; State Key Laboratory of Robotics and Systems, Harbin Institute of Technology, China; State Key Laboratory of Robotics and Systems, Harbin Institute of Technology, China

Since the pioneering deployment of Canadarm in 1981, various on-orbit servicing (OOS) concepts have been flourishing and are continuously demonstrated by space agencies worldwide [1]. This novel access to extend human curiosity in space is empowered by the remarkable OOS progress achieved by missions like the Robot Technology Experiment (ROTEX), Engineering Test Satellite-VII (ETS-VII), Orbital Express, Robotic Refueling Mission (RRM), Robonaut-2, RemoveDebris and Mission Robotic Vehicle (MRV). As a space-faring nation, China is also dedicated to developing OOS robots. The Shiyan-7 satellite, equipped with the first space robot of China, accurately captured a cooperative object in 2013 [2], making a pathfinder effort toward on-orbit manipulation in low Earth orbit (LEO). As a crucial stepping stone for establishing a modular space station with long-term human participation, the on-board human–robot collaboration was demonstrated in the Tiangong-2 space laboratory in 2016 [3]. The on-orbit assembly of the Chinese Space Station was scheduled to be completed in 2022, wherein two seven-joint manipulators with lengths of 10 m and 5 m could collectively carry out intricate tasks in either teleoperated or automated modes, e.g. module transposition, full-range payload care and support for astronaut extravehicular activities [4]. The Shijian-21 satellite, launched in 2021, enabled technological breakthroughs for alleviating space debris threats in geosynchronous orbit (GEO).

Current evolutionary trends of OOS tasks are showing rapid progression, from elementarily clamping cooperative LEO spacecraft via a single manipulator under master-slave teleoperation, to sophisticatedly manipulating uncooperative GEO spacecraft via multiple manipulators with physical telepresence.

Besides accomplishing the impressive on-orbit missions, substantial proof-of-concept investigations are ongoing:

- *Space debris removal.* It is a global concern that Earth orbits are gradually being filled with defunct spacecrafts and their collision fragments; however, a highly reliable method of capturing and detumbling those non-cooperative targets is still absent. There is an urgent need to improve robots’ perceptual capability while mitigating human operators’ cognitive burden, in the quest to improve system-wide autonomy as well as adaptability to diverse objects with unknown kinematic/kinetic properties.
- *Life extension services.* Life extension services for both existing and future spacecrafts provide a promising method of reinforcing orbital sustainability. Though a single attached tug has been validated to power the client satellite by Mission Extension Vehicle (MEV), integrating multiple tugs into a host spacecraft, analogous to the Payload Orbital Delivery (POD) system [5], is expected to accelerate delivery tempo. On-orbit refueling services may be enhanced by equipping the fuel and client vehicles with standard transfer interfaces, to cost-effectively establish an in-space propellant supply chain.
- *On-orbit assembly and manufacturing.* Given the volume limits imposed by rockets, high-precision robots substituting human astronauts are envisaged to execute in-space construction of large-scale antennae, telescopes and solar power stations. In order to carry out these delicate and dexterous assignments in the harsh space environment, it is anticipated that robotic technologies will be enhanced by high-performance hardware, intelligent control strategies and well-planned maneuver procedures.

Moreover, ground-based verification, which is required in order to simulate the true dynamics of six-degrees-of-freedom microgravity operation, remains challenging, especially for large-scale robotic systems. By addressing the aforementioned open issues, space robotics that integrate humans, robots and the space environment into a tri-co system will further streamline OOS missions, therefore deepening human insight into space and benefiting sustainable development.

## Conflict of interest statement

None declared.
